# Health information priorities and recommendations: findings from community listening sessions with women of refugee background from Myanmar in Western Australia

**DOI:** 10.1080/17482631.2026.2729863

**Published:** 2026-09-09

**Authors:** Georgia Griffin, S. Zaung Nau, Mohammed Ali, Elisha Riggs, Jaya Dantas

**Affiliations:** a Curtin School of Population Health, Curtin University, Bentley, WA, Australia; b School of Management and Marketing, Curtin University, Bentley, WA, Australia; c Policy and Equity, Murdoch Children's Research Institute, Parkville, VIC, Australia; d Department of General Practice and Primary Care, University of Melbourne, Parkville, VIC, Australia

**Keywords:** Communication barriers, health information, health promotion, Myanmar, refugees, women's health

## Abstract

**Purpose:**

Targeted evidence is needed to inform health promotion initiatives amongst growing Myanmar diaspora communities in Australia and globally. This study aimed to report on health information priorities and recommendations identified by women of refugee background from Myanmar settled in Western Australia and their female community leaders.

**Methods:**

Guided by participatory action research principles, four community listening sessions were conducted with 39 women of refugee background from Myanmar and community leaders in February and March 2024. Women were from Karen and Chin cultural groups living in metropolitan and regional communities. Data underwent content analysis informed by an unstructured analysis matrix.

**Results:**

Five categories and 27 subcategories were identified: 1) changing contexts, 2) motivations, 3) information priorities, 4) information sources, and 5) structural barriers for action. Categories contextualise women's health information experiences and priorities, before offering a set of topics for which women needed information. Identified gaps in available information resources and structural barriers have implications for health and settlement policies.

**Conclusions:**

Health literacy is framed as a collective, relational, and structurally constrained process for women of refugee background from Myanmar. To support women to build their health knowledge, comprehensive health promotion initiatives and targeted improvements to structural barriers are needed.

## Introduction

Myanmar, previously known as Burma, has undergone decades of political instability and violent conflicts, prompting waves of forced displacement and migration globally (Fike & Androff, 2016). Most recently, the 2021 military coup and subsequent civil war have contributed to this humanitarian crisis (United Nations High Commissioner for Refugees; United Nations Women, 2024). In mid-2025, approximately 1.5 million people were forcibly displaced from Myanmar (United Nations High Commissioner for Refugees Regional Bureau for Asia & the Pacific, 2025). Countries such as the United States and Australia have significant Myanmar diaspora populations, largely comprised of humanitarian entrants (Fike & Androff, 2016; Reid, 2024; Trieu & Vang, 2015). These populations are ethnically, culturally, and linguistically diverse, including Burmese, Karen, and Chin cultural groups (Fike & Androff, 2016; Griffin et al., 2022). In Australia, the Myanmar-born population has increased rapidly in the past two decades from 28,660 people in 2013 to 44,770 people in 2023 (Australian Government Department of Home Affairs, 2025; Reid, 2024). Myanmar was the third most common source country for humanitarian entrants to Australia in 2023–2024, of which approximately half were women.

In Australia and other settlement countries, women of refugee background from Myanmar face complex intersecting challenges when both accessing and navigating health services (Griffin et al., 2022). Identified barriers include language discordance, inconsistent provision of interpretation, fragmented healthcare systems, transport and difficulty locating services, financial costs, and competing gendered settlement priorities such as caring for children (Griffin et al., 2022a; Griffin et al., 2022b; Wong et al., 2024). For refugee background communities in regional Australia, barriers related to transport and distance to services can be exacerbated, along with additional barriers related to accessibility, affordability, and cultural responsiveness of available health services, lack of health services, limited provision of interpreters, and poor availability of multilingual health information resources (Khatri & Assefa, 2022; Mude, 2026). Limitations to functional health literacy, essential skills enabling women to obtain and apply health information (Nutbeam & Lloyd, 2021), can also pose a barrier. Previous research has also reported on the resilience of refugee-background populations from Myanmar, drawing on community networks to overcome barriers to accessing services (Griffin et al., 2022a, 2022b; Wong et al., 2020, 2024).

Health literacy and health information play a pivotal role in navigating health services and promoting good health (Nutbeam & Lloyd, 2021). Prior studies with other refugee-background populations have explored health literacy as socially, culturally, and structurally situated (Chao & Kang, 2020; Peprah et al., 2025; Samerski, 2019). From an ethnography with people of refugee background from Bhutan residing in the United States, Chao and Kang (2020) found that health literacy was constructed, shared, and developed through relationships and conversation with one another. Health information and knowledge were similarly found to be distributed and shared across social networks and relationships in a qualitative exploration of health literacy amongst Africans of refugee background residing in Australia (Peprah et al., 2025). The authors similarly concluded that health literacy among people of refugee background from Africa was a context-specific social practice, influenced by health services and provider processes. In both studies, health literacy was primarily shaped by oral interactions with trusted people within social networks, including family members, well positioned as health literacy brokers and co-producers of knowledge. Amongst refugee background communities from Myanmar, a collective element to health has also been identified (Wong et al., 2020). A qualitative exploration of the health information experiences of women of refugee background from Myanmar living in Western Australia (WA) highlighted community networks as a salutogenic resource for women and health information seeking as a collective effort (Griffin et al., 2022). These studies suggest that health literacy may similarly be socially and culturally constructed amongst this population.

Previous studies have also explored influences on health, health literacy, and health information experiences of refugee-background populations from Myanmar during settlement in Australia (Davidson et al., 2024; Griffin et al., 2022a; *Wong et al*., 2020, 2024). One qualitative study reported that refugee-background people from Myanmar valued their health, viewing it holistically as inclusive of physical, mental, and social wellbeing (Wong et al., 2020). In addition to community networks, internet-based sources, schools, settlement services, and healthcare providers have been identified as sources of health information for women of refugee background from Myanmar in WA (Davidson et al., 2024; Griffin et al., 2022a). Healthcare providers specifically have been highlighted as primary and preferred information sources for women, reflecting pre-migration experiences (Davidson et al., 2024; Griffin et al., 2022a; Wong et al., 2024). Motivation to access a healthcare provider has been associated with symptoms of ill health or pregnancy (Davidson et al., 2024; Griffin et al., 2022a). Reported health information needs have included healthy eating and nutrition (Wong et al., 2020, 2024), exercise (Wong et al., 2020), and sexual and reproductive health (SRH) (Wong et al., 2020), including pregnancy (Griffin et al., 2022a) and breast and cervical cancer screening (Davidson et al., 2024).

Multiple national and state government policies inform the availability and accessibility of health information for women of refugee background from Myanmar in WA, along with other refugee-background populations across Australia (Commonwealth of Australia & Department of Health, 2026; Department of Health & State of Western Australia, 2024; Western Australian Department of Health, 2019) such as the National Preventive Health Strategy 2021–2030 (Commonwealth of Australia & Department of Health, 2026). These policies outline strategies to promote health and wellbeing and identify culturally and linguistically diverse, or migrant and refugee, communities as priority groups. Similarly, national settlement policies provide structural direction across all levels of government to support new migrants, with health and wellbeing highlighted as a priority area (Australian Government Department of Home Affairs, n.d.; Australian Government Department of Home Affairs, n.d.). Despite these cross-sector policy commitments, there is limited evidence about the health information priorities of women of refugee background from Myanmar or how health information and promotion initiatives can be designed to respond to their health literacy practices and information needs. This lack of evidence limits the ability of health services and policymakers to develop responsive, community-informed health promotion strategies. This study aimed to report the health information priorities and recommendations identified by women of refugee background from Myanmar settled in WA and their female community leaders.

## Materials and methods

### Study design

This article reports on one study conducted within a broader multi-method research project exploring the health information needs of women of refugee background from Myanmar living in WA (Griffin et al., 2022a, 2022b). Participatory action research (PAR) principles underpinned the project, privileging collaboration, knowledge exchange and reciprocity with women and their communities towards social change (Vallianatos et al., 2015). An earlier study within this project explored women's health information experiences and developed a model describing women’s experiences (Griffin et al., 2022a). Consistent with the iterative nature of PAR, these study findings informed the present study design, providing a foundation for progression from the earlier exploration of women's experiences towards identifying priorities and recommendations with female community members and leaders.

A stakeholder reference group provided feedback on the study design, recruitment strategies and preliminary findings. Members of this group include people with experience in community leadership, interpretation, policy, and service provision. Aligned with PAR, an intersectional lens guided the research team to remain responsive to intersecting factors influencing women's health information experiences or engagement with the project (Couto et al., 2019; Higginbottom & Liamputtong, 2015).

### Setting

This study was conducted in two cities in WA, the capital city, Perth, and a regional centre, Albany. Humanitarian entrants typically arrive in Perth, in which most health and settlement services are located (North Metropolitan Health Service, 2025) or are placed in regional areas with comparably limited access to health services (Australian Government Department of Home Affairs, 2024). They have access to specific settlement support services up to five years post-arrival (Australian Government Department of Home Affairs, 2025). Australian health and settlement services have access to government-funded national interpretation and translation services (Australian Government Department of Home Affairs, n.d.). While in-person interpreters can be accessible in Perth, most interpreters are accessed via telephone or video in regional settings.

### Participants and sampling

Women were eligible to participate if they identified as of refugee background from Myanmar or as female community leaders within the Myanmar diaspora living in WA and were 18 years or older. Purposive and snowball sampling techniques were employed (Bekteshi et al., 2024). The research team partnered with community-based organizations who worked with Myanmar diaspora women or communities. Karen and Chin language flyers advertising the sessions were disseminated through these organizations to diaspora communities. Women were encouraged to share the flyers and bring other eligible women.

Participant recruitment was informed by community organizations' knowledge of the communities they worked with. The sample size reflected community co-convener's access to these communities, community interest in participation, community co-convenor availability to support data collection, and available study funding. Information power was also a consideration. Following each session, the first author reviewed session field notes, observing new and recurring concepts. This was not a formal assessment of data saturation; however, this process informed decisions to continue participant recruitment in conjunction with the guidance from community organizations described.

### Data collection

Community listening sessions were selected as a distinct data collection approach theoretically informed by focus group discussions, learning circles, and participatory research (Ardoin et al., 2022). They are flexible in structure and privilege group discussions as a source of collaborative knowledge exchange (Vallianatos et al., 2015), ideally suited for PAR with underrepresented communities (Erves et al., 2017; Walker et al., 2022). Community listening sessions are not only designed to elicit individual participant perspectives in response to facilitator questions, but also to capture new understandings generated through group discussion of concepts and collaborative construction of ideas and potential solutions (Ardoin et al., 2022). Logistical considerations include collaboration with community partners in both session planning and facilitation, convening of session participants already known to one another, selection of a location familiar to participants, room layout designed to reduce hierarchy or power differentials, e.g., chairs positioned in a circle, along with other socio-cultural considerations to promote comfort.

Sessions were held in community venues in locations with known Myanmar diaspora populations. A female interpreter was arranged by video-conference in Albany and in-person in Perth. An English-speaking facilitator, GG, led each session in collaboration with a community co-convener, typically a representative from a community-based organization known to the community, present for some or all of each session to promote trust and reciprocity.

On arrival to each session, women completed a brief anonymous language-concordant demographic questionnaire. A semi-structured question guide, informed by the earlier exploration of women's health experiences (Griffin et al., 2022a) and a facilitation plan were used for each session. Data collection materials are presented in the supplementary material. Session questions explored what good health means to women, the role of health information, health information priorities and sources, and recommendations for service providers and policymakers. To enact PAR principles of reciprocity and collaborative knowledge exchange, the laminated model of health information experiences developed in the earlier study (Griffin et al., 2022a) was also presented. This created an opportunity for participants to refine, extend, or challenge these pre-existing understandings, aligning with the broader participatory approach.

Women were asked to draw or write in their preferred language in response to questions, following which responses were discussed with the group. To meet the study aim of identifying women's priorities, where appropriate to the question, responses were displayed, discussed, and once consensus was reached through group agreement, ranked in order of importance. For some questions, participants agreed through discussion that multiple or all responses were of equal importance. For other questions, participants agreed that there was an order of priority to their responses. Photos were taken of displayed responses and rankings. Written and drawn responses were also collected for data analysis. Two sessions were audio-recorded with consent. Two sessions were not audio-recorded at the participants' preference. Field notes were taken during and following all sessions by the facilitator.

Following each session, the facilitator (GG) engaged in informal conversations with the community co-convener, reflecting upon what was shared in each session and participant engagement in dialogue. GG was an outsider to both the women's communities and organizations supporting the sessions, and an insider to the health sector as a nurse midwife. Given this positionality, these conversations supported the first author's reflexive examination of her perceptions of and reactions to the data collection in each session (Arriaza et al., 2015).

Prior to each session, language-concordant participant information sheets and consent forms were provided and discussed. Sessions were only audio-recorded if all participants consented to the recording. Each participant was offered a $25 AUD grocery voucher as an honorarium.

### Data analysis

S'Gaw Karen and Chin Hakha's written and audio data were professionally translated and transcribed into English. The English-language audio data were also transcribed by GG. One written participant response was removed for analysis because multiple translators were unable to identify the language. Content analysis was conducted on written participant responses, descriptions of photographs and drawings, transcripts, and field notes, following the phases outlined by Elo and Kyngäs (2008). These were preparation, organizing, and reporting.

A deductive approach was initially taken, using an unstructured analysis matrix informed by the pre-existing model of women's health information experiences (Griffin et al., 2022a). An unstructured analysis matrix was selected to allow space for the inclusion of findings that did not fit into the matrix (Kyngäs & Kaakinen, 2020), enabling refinement, extension, or challenge of pre-existing knowledge generated through the earlier study (Griffin et al., 2022). The initial matrix included five deductive categories reflecting select components of women's health information experiences: *Motivation*, *Obtaining*, *Understanding*, *Appraising*, and *Using Information*. Data from one session were independently inductively coded by GG and MA. Coding was compared and discussed to explore alternative interpretations (Graneheim et al., 2017). The codes were organized into the matrix and the fit was discussed by the two researchers, referring to the research aim to report health information priorities and recommendations. An abductive approach was then taken to revising the matrix, allowing for the findings to extend upon this foundation. The revised matrix was discussed and agreed upon by GG and MA. The full dataset was then manually inductively coded by GG and organized into categories defined in the matrix.

Categories and inductive sub-categories were agreed upon as the highest level of abstraction for analysis. Given the knowledge exchange and collaborative construction of ideas and solutions inherent to community listening sessions (Ardoin et al., 2022), data analysis considered both individual perspectives elicited, along with the collaborative understandings generated through group discussion. To promote trustworthiness, preliminary findings were presented to the stakeholder reference group, facilitating interpretation and confirmation of the findings. Throughout the data analysis process, GG made reflexive notes, critically engaging with her positionality and the influence of this on analysis (Olmos-Vega et al., 2023).

Demographic data underwent descriptive statistical analysis. Frequencies, percentages, medians, and ranges were calculated for the total sample. Demographic and qualitative findings are reported by setting, metropolitan or regional, below. This reflects the variation in access to health services and in-person interpreters between metropolitan and regional WA (Australian Government Department of Home Affairs, 2024).

## Results

Four community listening sessions were conducted in February and March 2024. Sessions lasted two to 2.5 hours in length including a refreshment break. Seven to fifteen women attended each session, providing a total of 39 participants.

### Participant characteristics

Participant characteristics are presented in Table I by setting, metropolitan or regional. Participants were aged 27 to 78 years (median 42 years) and had arrived in Australia between 1968 and 2023 (median 2013). Of the total 39 women, most reported speaking Karen (*n* = 23, 59.0%) or Chin (*n* = 15, 38.5%) languages. All regional participants spoke Karen. Most women reported reading (*n* = 33, 84.6%) and writing (*n* = 31, 79.5%) well or very well in their primary language. In contrast, most reported reading (*n* = 30, 76.9%) and writing (*n* = 33, 84.6%) not well or not at all in English. All regional participants required an interpreter for health appointments sometimes (*n* = 3, 18.8%) or always (*n* = 13, 81.3%), whereas 21.7% required an interpreter sometimes (*n* = 5) and 56.5% always required an interpreter (*n* = 13).

Most participants were parents or carers for children (*n* = 33, 84.6%), including all regional participants. Only 7.7% of participants were employed full-time (*n* = 3), all located in the metropolitan setting. Of those living regionally, most were homemakers (*n* = 7, 43.8%) or in part-time employment (*n* = 4, 25.0%). Most metropolitan participants reported part-time employment (*n* = 6, 26.1%), unpaid work such as volunteering (*n* = 6, 26.1%), and identified as homemakers (*n* = 5, 21.7%). Other responses to employment status included unpaid study and receiving government assistance. Most frequently, women's highest level of education was technical and further education (TAFE) (*n* = 19, 48.7%), such as English language classes. Approximately one third of participants had not had any schooling (*n* = 4, 10.3%) or attended primary school only (*n* = 9, 23.1%).

**Table I.** Participant characteristics by setting.

	Metropolitan *N* = 23	Regional *N* = 16	Total *N* = 39
Age in years, median (range)	41 (27–64)	43.5 (30–78)	42 (27–78)
Year arrived in Australia, median (range)	2012 (1968–2023)^#^	2016 (2005–2019)	2013 (1968–2023)^#^
*Primary language spoken (n, %)* *			
Karen	7 (30.4)	16 (100.0)	23 (59.0)
Chin	15 (65.2)	0 (0.0)	15 (38.5)
Burmese	2 (8.7)	0 (0.0)	2 (5.1)
English	2 (8.7)	0 (0.0)	2 (5.1)
*Reading proficiency in primary language (n, %)* ^a^			
Very well	16 (69.6)	11 (68.8)	27 (69.2)
Well	4 (17.4)	2 (12.5)	6 (15.4)
Not well	1 (4.3)	1 (6.3)	2 (5.1)
Not at all	2 (8.7)	1 (6.3)	3 (7.7)
*Writing proficiency in primary language (n, %)* ^a^			
Very well	15 (65.2)	10 (62.5)	25 (64.1)
Well	4 (17.4)	2 (12.5)	6 (15.4)
Not well	3 (13.0)	2 (12.5)	5 (12.8)
Not at all	1 (4.3)	1 (6.3)	2 (5.1)
*Reading proficiency in English (n, %)* ^a^			
Very well	1 (4.3)	0 (0.0)	1 (2.6)
Well	3 (13.0)	3 (18.8)	6 (15.4)
Not well	10 (43.5)	6 (37.5)	16 (41.0)
Not at all	8 (34.8)	6 (37.5)	14 (35.9)
*Writing proficiency in English (n, %)* ^a^			
Very well	1 (4.3)	0 (0.0)	1 (2.6)
Well	2 (8.7)	2 (12.5)	4 (10.3)
Not well	12 (52.2)	8 (50.0)	20 (51.3)
Not at all	8 (34.8)	5 (31.3)	13 (33.3)
*Interpreter required for health appointments (n, %)*			
Yes, always	13 (56.5)	13 (81.3)	26 (66.7)
Yes, sometimes	5 (21.7)	3 (18.8)	8 (20.5)
No	5 (21.7)	0 (0.0)	5 (12.8)
*Parent or carer (n, %)* ^a^			
Yes	17 (73.9)	16 (100.0)	33 (84.6)
No	4 (17.4)	0 (0.0)	4 (10.3)
*Employment status (n, %)* *			
Paid work full-time	3 (13.0)	0 (0.0)	3 (7.7)
Paid work part-time	6 (26.1)	4 (25.0)	10 (25.6)
Paid study/scholarship	1 (4.3)	1 (6.3)	2 (5.1)
Unpaid work (e.g. volunteering)	6 (26.1)	0 (0.0)	6 (15.4)
Homemaker	5 (21.7)	7 (43.8)	12 (30.8)
Other	1 (4.3)	5 (31.3)	6 (15.4)
*Highest level of education (n, %)*			
No schooling	3 (13.0)	1 (6.3)	4 (10.3)
Primary school	4 (17.4)	5 (31.3)	9 (23.1)
High school	0 (0.0)	3 (18.8)	3 (7.7)
TAFE	13 (56.5)	6 (37.5)	19 (48.7)
University degree	3 (13.0)	0 (0.0)	3 (7.7)
Other	0 (0.0)	1 (6.3)	1 (2.6)

^a^ Valid percentages reported.

^#^ Year of arrival missing for one participant.

^*^ Multiple responses selected.

### Health information priorities and recommendations

**Figure 1.**
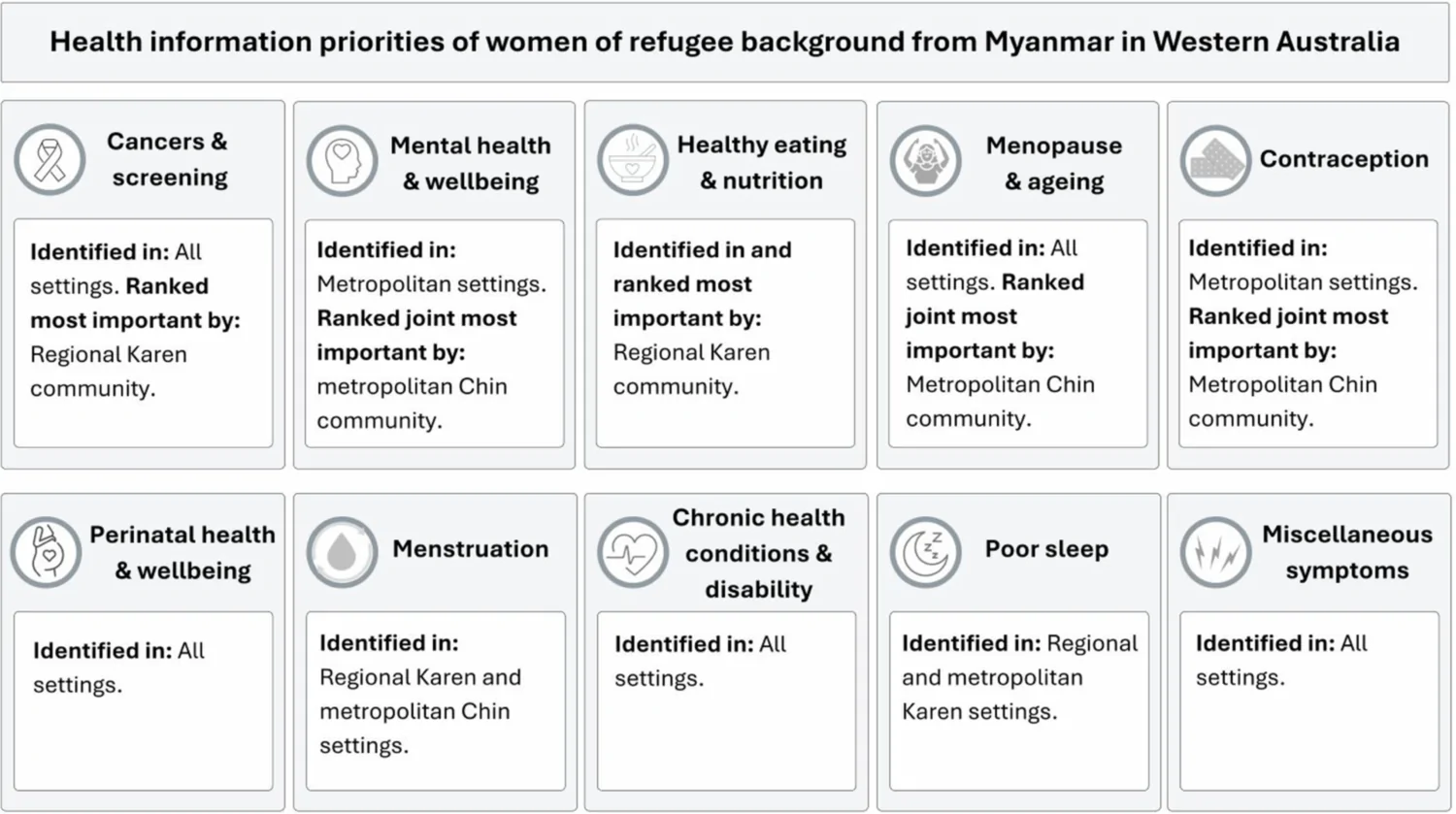
Health information priorities identified by women of refugee background from Myanmar and female community leaders in WA.

**Figure 2.**
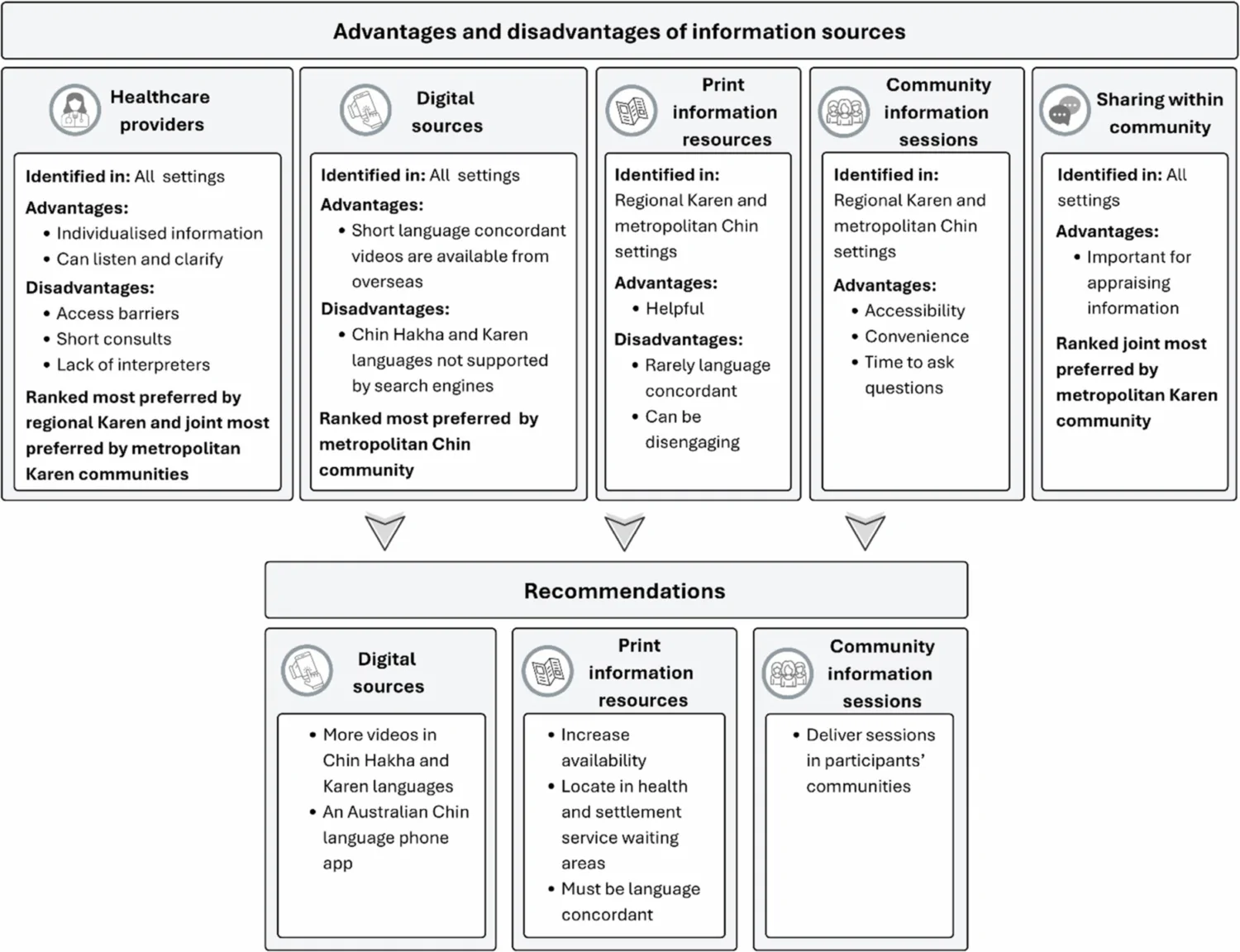
Rankings of and recommendations for health information sources as identified by women of refugee background from Myanmar and female community leaders in WA.

**Figure 3.**
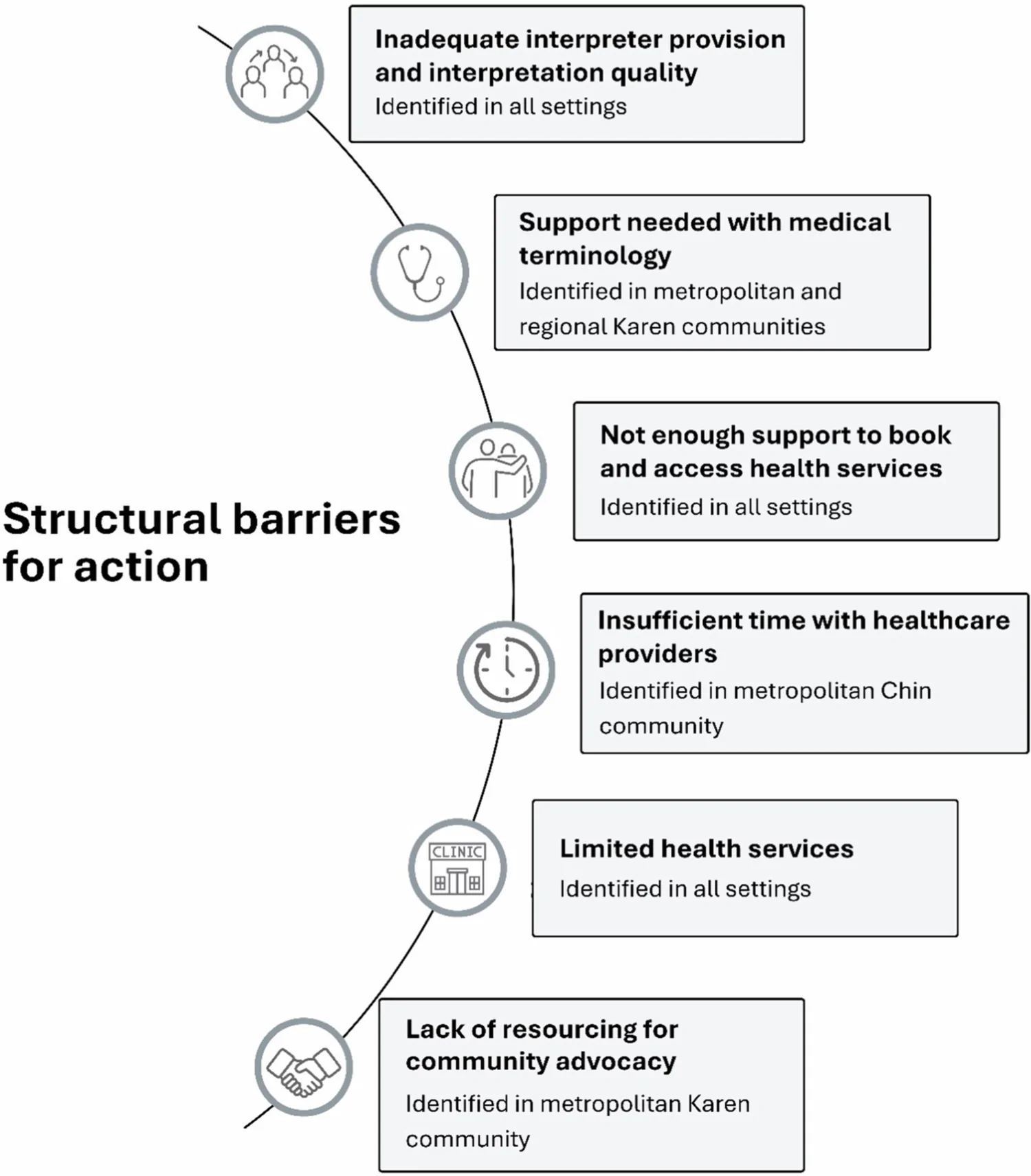
Structural barriers identified for action by women of refugee background from Myanmar and female community leaders in WA.

Deductive content analysis resulted in five categories and 27 subcategories. Categories were: 1) Changing contexts, 2) Motivations, 3) Information priorities, 4) Information sources, and 5) Structural barriers for action. Figures 1–3 present the latter three categories and associated subcategories. Categories and subcategories are also described below with supporting quotes. Each quote is followed by ethnic group and setting of the session: Chin or Karen; Metropolitan or Regional.

#### Category 1. Changing contexts

For service providers and policymakers to understand their priorities and recommendations, participants explained that they must understand the changing contexts of women's lives pre- and post-settlement in Australia. Subcategories were Prior healthcare experiences, Collective cultures, and Gendered responsibilities.

##### Subcategory 1.1 Prior healthcare experiences.

Participants' pre-settlement healthcare experiences were characterized by a focus on survival, limited resources, and poor treatment by doctors in Myanmar, and good healthcare access and treatment by doctors in refugee camps. Pre-migration, the focus was on survival, not health: “*we don't have time to think about health because it's a war zone*” (Karen, metropolitan). Many lived without healthcare access or clean water and were treated poorly by doctors in Myanmar. In contrast, in the refugee camps, Karen participants described ready access to a doctor, queries answered, and assistance provided to determine the urgency of their health needs. Doctors were described as having knowledge of and respect for Karen culture.

##### Subcategory 1.2 Collective cultures.

In this subcategory, Karen participants' juxtaposed descriptions of pre-settlement lifestyles reflective of a collective culture, with post-settlement challenges to supporting one another. They described helping each other and sharing in each other's lives pre-settlement. Although participants continued to help each other during settlement in Australia, their lifestyles had changed. For example, traditional medicines were no longer always accessible, and blessings were no longer believed to cure illness. Husbands were busy working, and the cost of living was high. As one participant explained, no one was going to care for women but themselves, “*if we are not healthy while living in another country like this, our husbands may be too busy to take care of us. We have lots of expenses here, so it's important to good care of ourselves*” (Karen, regional).

##### Subcategory 1.3 Gendered responsibilities.

Participants described gendered caregiving roles which could exacerbate mental health stressors, while the Church was framed as protective. There was a gendered emphasis on women caring for their children, husbands, and families. These responsibilities could isolate women in the home with an impact on their mental health and health information experiences acknowledged by the women themselves. Church was presented as a link to community and connection, “*women are always at home looking after their children, we are always at home, for some people this can cause mental illness… when we attend the Church our heart is quite freshen [refreshed]*” (Chin, metropolitan). Trauma from pre-settlement experiences and troubling current news about Myanmar could also threaten women's ability to meet these gendered responsibilities, “*we have our own families, and if we watch [news about Myanmar] that affect our health, it can impact us*” (Karen, regional).

#### Category 2. Motivations

Participants described motivations for seeking out and engaging with health information and healthcare. Subcategories were Towards good health and Worries about ill health.

##### Subcategory 2.1 Towards good health.

Participants valued good health for what it enabled them to do and how they felt when they were healthy. Emphasis was placed on what being healthy enabled women to do—be active, work, care for family, go out of the home and connect with friends and family. Sleep, healthy food, exercise, and regular medical checkups, particularly cervical screening tests, were identified as factors in good health.

“*To have a happy and healthy life with smiling face and singing, we must express our love and showing interest toward each other, give us peaceful heart and satisfaction. We must have this kind of health… If we worry too much, it will affect our health*” (Karen, regional).

Health information was recognized as a facilitator of good health, “*accessing health information advises and warns us what to do and what not to do for our health*” (Chin, metropolitan).

##### Subcategory 2.2 Worries about ill health.

Participants' motivations also reflected a perceived responsibility to be healthy for their families and fear of ill health. They were motivated by fears of illness, particularly cancers, to seek good health. Participants wanted to know about screening for ill health and how to prevent illnesses suffered by others in their communities, “*I want to know what sort of diseases are in my body. In our community, we do not see the doctors often. For example, we have no idea what diseases are in our body*” (Karen, regional). Seeing a doctor for screening and information was therefore a source of reassurance for these fears, “*after getting a proper check-up, we feel relief because we know that our body is healthy*” (Chin, metropolitan). However, a lack of knowledge about feared illnesses persisted, reflected in women's health information priorities presented in Category 3.

Not all women were motivated by their fears to engage with healthcare providers; however, for some, fear could inhibit engagement, “*when I hear anything about health, I become very anxious*” (Karen, regional).

#### Category 3. Information priorities

Women identified health information priorities for women in their communities and described their own knowledge gaps. These were grouped into subcategories presented in Figure 1. Subcategories identified as most important in one or more sessions were Cancers and screening, Mental health and wellbeing, Healthy eating and nutrition, Menopause and ageing, and Contraception. Further identified subcategories were Perinatal health and wellbeing, Menstruation, Chronic health conditions and disability, Poor sleep, and Miscellaneous symptoms. Subcategories are described below.

##### Subcategory 3.1 Cancers and screening.

Cancer and cancer screening were identified as a priority in all settings. As shown in Figure 1, regional Karen participants ranked cancer and cancer risk as the most important information need for women in their community, “*the cancer is the most important because what we eat and what we do can affect it and if people don't know about it, then they cannot prevent it and they will lose their life quite quickly*” (Karen, regional). Participants wanted information about assessing individual risk, prevention, screening, signs and symptoms, and treatment options. They had a particular focus on uterine, cervical and breast cancers, reflected in requests for information about, “*how to take care of uterus health*” (Chin, metropolitan). Many were unaware or unsure that other cancer types existed.

##### Subcategory 3.2 Mental health and wellbeing.

Mental health and wellbeing were identified as a priority in metropolitan settings only. Information needs specific to symptoms of anxiety and depression, post-traumatic stress and problems with healthy relationships were described. It was ranked by Chin participants as one of the most important information needs for women in their community, “*mental health is important is like we can do everything if our mental health is good and we can't do anything if our mental health is not well*” (Chin, metropolitan). Mental health was positioned as a community problem because, as one participant explained, “*psychological health affects people around me*” (Karen, metropolitan).

##### Subcategory 3.3 Healthy eating and nutrition.

Healthy eating, maintaining a healthy weight, nutrition and vitamin intake were also discussed in every session, although only regional Karen participants identified this as a priority. They ranked diet as their most important community information need to give them energy. Food and nutrition were also associated with preventing weakness, enhancing strength, and preventing cancer. Many participants reported “*poor appetite*” (Karen, regional). Women reported an absence of information about nutrition specific to traditional foods and expressed uncertainty about whether vitamin supplements would help them to feel stronger. One participant explained,

“*no one knows how to eat accordingly and enough. We need vitamins, and we want to buy them over the counter, but we do not know how to read English properly. We do not know what is good and better for us to eat*” (Chin, metropolitan).

##### Subcategory 3.4 Menopause and ageing.

Maintaining good health in the context of menopause and ageing was identified as an information need in all settings. Chin participants ranked menopause as one of the most important information priorities for women in their community. Information about symptoms, prevention, treatment and management options, and indications for treatment was needed. There was also a focus on promoting strength during midlife and as they aged,

“*every woman over the age of 40 knows that her period will end anyway. We are ready to take care of ourselves, including what the doctor tells us. I would like to know more about how we can prevent it safely or is there any specific way to do it*” (Chin, metropolitan).

Diet and exercise were also identified as important for maintaining health as women aged beyond menopause. Regional Karen participants reported a need for information about “*exercise for older people—how to stay healthy, strong, active, keep balance*” (Karen, regional).

##### Subcategory 3.5 Contraception.

Chin women identified contraception as an information need and ranked it as one of their most important community information needs. Participants articulated a need for information to support decision-making about contraception, namely types of contraception, how each should be used, and respective advantages and disadvantages. One participant explained, “*sometimes we do not know much about the information, especially the side effects and some of the women who put contraception in her arm have a lot of physical problems… we want to know more information on it*” (Chin, metropolitan). They also expressed community concerns about social impacts, “*I have heard that when someone put contraception, there are a lot of problem between family members and couples*” (Chin, metropolitan).

##### Subcategory 3.6 Perinatal health and wellbeing.

Perinatal health and wellbeing were identified as community information needs in both settings. Regional Karen participants wanted information about being pregnant later in life and the associated risk of Down Syndrome while Chin participants wanted information about pregnancy and fertility more broadly. Karen participants also reported a community need for more postnatal information, “*mother and newborn health, such as how mothers with young babies can take care of themselves both mentally and physically*” (Karen, regional). Regional Karen participants also emphasized a need for information about perinatal depression.

##### Subcategory 3.7 Menstruation.

Menstruation was identified as an information priority for Chin and regional Karen participants. Information was needed about normal menstruation, management of pain and wellbeing, and menstruation after giving birth. One participant explained information was needed about “*managing periods, normal periods and period pain*” (Karen, regional). Another emphasized, “*women have period… we need to take care of ourselves*” (Karen, regional).

##### Subcategory 3.8 Chronic health conditions and disability.

Participants in all settings identified chronic health conditions about which women in their community needed more information. These included asthma, eye problems, kidney disease, liver health including hepatitis, diabetes, haemorrhoids, and high blood pressure. Chin participants ranked haemorrhoids specifically as one of their most important priorities. Participants also wanted information about services for chronic conditions, “*diabetes, high blood pressure… who to see for these problems*” (Karen, regional).

Participants similarly reported a need for information about “*NDIS/disability*” (Karen, regional) services, including what the National Disability Insurance Scheme (NDIS) is. Participants explained that few women sought information about or assistance with disabilities because they had become used to managing without support.

##### Subcategory 3.9 Poor sleep.

Sleep was identified as an information need by Karen participants in both settings. This aligned with sleep identified as an important aspect of good health in Category 2. They wanted information about sleeping well in the context of ageing, improving sleep, and causes of poor sleep. One participant explained, for example, “*I think I do not sleep well because of my age; what shall I do to have a good sleep?*” (Karen, regional).

##### Subcategory 3.10 Miscellaneous symptoms.

In all settings, participants described symptoms of ill health of unknown cause, most frequently pain and skin ailments. They confirmed these were community-wide concerns. They wanted information about causes of symptoms and how to resolve them. Commonly reported types of pain included headaches, chest tightness, and abdominal pain, “*sore hands, sore feet, and joint pain, it happens more now. What shall I do?*” (Karen, regional). Skin ailments included eczema, pigmentation, “*rashes, dry skin, itchy skin*” (Karen, regional).

#### Category 4 Information sources

Participants described types of information sources, some pre-existing and some needed, grouped into subcategories. Presented in Figure 2, subcategories were Healthcare providers, Digital sources, Print information resources, Community information sessions, and Sharing within community. Participants reflected on the availability, advantages, and disadvantages of each, and made recommendations for more and improved resources.

##### Subcategory 4.1 Healthcare providers.

Healthcare providers were a frequently identified source of information in all settings. These primarily included general practitioners or doctors more generally. Regional Karen participants ranked obtaining information from a knowledgeable person as their preferred information source, while metropolitan Karen participants ranked health services, when interpreters were provided, as their joint most preferred information source. For some, due to language and literacy barriers, GPs were viewed as the only accessible information source, although in one session, the local settlement service provider was also identified as a source of health information. Participants explained that once women gained experience accessing health information from a doctor, they could continue to seek out that doctor when they needed information.

GPs were valued because they could explain information specific to women's circumstances and women could clarify information obtained from elsewhere: “*you can listen and clarify*” (Karen, regional). Participants confirmed that they felt confident to ask questions to appraise information and apply it to their own context. However, structural barriers outlined in Category 5 could inhibit effective communication of health information. Chin participants explained, “*just going to the GP alone is not enough for the overall matter of our health*” (Chin, metropolitan). Acting upon health information was another challenge: “*when a doctor gives me instructions or the blood test shows my cholesterol is so high, I try to control my diet and avoid fatty food. We know it but we cannot apply it*” (Chin, metropolitan). This sentiment, that applying the information was the hardest aspect of health literacy, was echoed amongst Chin participants.

##### Subcategory 4.2 Digital sources.

Digital sources were described in every session, specifically searching for online information using the Google search engine, short videos on social media platforms and a health information app. For some, “*Google is only good for looking up addresses*” (Chin, metropolitan), largely because it could only be used in English or Burmese. For others however, Google was more useful, *“sometimes, instead of going to the doctor for advice, we rely on information from Google and YouTube*” (Karen, regional).

Participants also reported watching brief health information videos created overseas and delivered in Chin Hakha or Karen on YouTube or Facebook. Chin participants ranked these as their preferred health information source while regional Karen participants described them as their second most preferred. Videos were valued because they were short, language-concordant, and women could search for specific topics. As highlighted in Figure 2, participants wanted more language-concordant health information videos; they were not aware of pre-existing videos made in Australia.

Chin participants advocated for the creation of an Australian Chin language health information phone app, “*the good thing about this app will be, for example, if we want to know about… contraception, weight loss, or mental health… we will just click on the app button and can easily find what we want*” (Chin, metropolitan). One participant described using an equivalent American app to access health information. While this was useful, participants wanted information appropriate to the Australian context.

##### Subcategory 4.3 Sharing within community.

Participants described exchanging health information within their communities in all sessions. They described sharing knowledge of traditional medicines and information gained from GPs previously, “*after we hear about the information, then it's helpful, we also want our friends to hear about it*” (Karen, regional). Metropolitan Karen participants ranked friends and family their joint most preferred source and Chin participants ranked them as their second most preferred information source. Discussing information with family was also important for appraising information, “*if I want to buy good medicine, firstly I ask the family members from other places and then only I keep on buying*” (Chin, metropolitan).

##### Subcategory 4.4 Print information resources.

Print information resources were identified in Chin and regional Karen settings as an information source that women would like but had seen little of. Regional Karen participants described brochures and posters as “*helpful but I think it should be in Karen language for us because English will not be helpful*” (Karen, regional). Some participants recalled print resources in Karen; however, they were uncommon, and more were needed. In contrast, Chin participants reflected that women in their community “*will be bored reading it even if it is released in our own languages*” (Chin, metropolitan), although they believed more language-concordant print information resources should be accessible to their community.

##### Subcategory 4.5 Community information sessions.

Community information sessions were highlighted by Chin and regional Karen participants as a desired information source. Participants did not describe attending any such sessions previously. In juxtaposition to identified barriers to healthcare providers, community information sessions were valued for accessibility, convenience and time to ask questions. Women anticipated that they could be held in familiar locations such as community churches. One participant explained, “*it will be good if they could provide health information through trainings, workshops and seminars in the church*” (Chin, metropolitan). Another explained the value of a community-based group setting in which health information was provided was that, “*we can take more time. I believe seeing doctors costs more, so attending a programme like this is the best option*” (Karen, regional).

#### Category 5. Structural barriers for action

Women identified structural barriers within their own health information experiences and those faced by other women in their communities. When asked to identify recommendations for service providers and policymakers, they highlighted these areas for improvement. Structural barriers, presented in Figure 3, were grouped into six subcategories: Inadequate interpreter provision and interpretation quality, Support needed with medical terminology, Not enough support to book and access health services, Insufficient time with healthcare providers, Limited health services, and Lack of resourcing for community advocacy.

##### Subcategory 5.1 Inadequate interpreter provision and interpretation quality.

The most frequently identified areas for improvement, highlighted in every session, were related to language barriers and interpretation. Regional Karen participants described language and lack of interpretation support as their biggest barrier to information. Reflecting the central role of healthcare providers, improved interpreter provision was recommended in GP and hospital settings. Excessive waits for healthcare providers to secure an interpreter were described, while at other times, interpreters were not provided at all: “*sometimes interpreter can be hard to access, you go to the service provider in the morning, they try in the morning and cannot, so you have to wait*” (Karen, regional).

Participants also recommended providing interpreters in their preferred languages, not Burmese: “*most of the time they get Burmese interpreter not Karen*” (Karen, metropolitan). Inconsistent interpretation quality was also an area for improvement. Some interpreters could inhibit communication with healthcare providers, “*when it's not clear and we ask them to explain again, they scold us, saying, just listen to me and answer yes or no*” (Karen, regional). Regional Karen participants highlighted interpretation as a barrier to appraising information in conversation with GPs. In the metropolitan setting, it was recommended that healthcare providers “*double check if women really understand well enough for consult to avoid miscommunication*” (Karen, metropolitan).

##### Subcategory 5.2 Support needed with medical terminology.

Medical terminology was also identified by regional Karen participants as a barrier to all aspects of their health information experiences, from seeking out information to using it. Participants described having to memorize English medical terminology: “*she has to remember the word cancer*” (Karen, regional). This was echoed by metropolitan Karen participants who explained that, for women in their community, not knowing the English words for their health concerns and how to write those words made it difficult to seek out information. The act of participating in a community listening session was perceived as an opportunity to learn some English medical terminology.

##### Subcategory 5.3 Not enough support to book and access health services.

When asked what they wanted service providers and policymakers to know, participants from all sessions wanted them to understand that they needed help to access health services, “*so for us to obtain [information] we will need help. Before obtaining, helps come first…, for example we don't have anyone to help us get to the doctor, then we cannot obtain the health information*” (Karen, regional). This included support to make appointments and with transport, which regional Karen participants identified as a barrier specifically. Informal support was valued, primarily accessed through children and other community members, and, in regional settings, through a trusted settlement service provider. Waiting for adult children and grandchildren to take time off work was a barrier between recognizing a health information need and acting to obtain information. There was also a recognized financial cost to helping one another to navigate barriers due to travel expenses for example, which added to the costs of appointments themselves: “*seeing doctors costs more*” (Karen, regional). The cost of health services in the context of the high cost of living was identified as the second biggest barrier to information by regional Karen participants.

##### Subcategory 5.4 Insufficient time with healthcare providers.

Chin participants recommended longer GP appointments. Short appointment length limited the amount of information women were able to obtain from GPs and prevented them from asking questions: “*the GP often says it does not have time to tell us more about such things in detail, so it cannot give us much information*” (Chin, metropolitan). Another participant explained how short appointment length inhibited communication,

“*we do not have time to talk to the GP about our illness in detail, the time he gives us is too short to explain and share about our illness, therefore, I wish he could extend the time to share and explain*” (Chin, metropolitan).

##### Subcategory 5.5 Limited health services.

Regional and metropolitan Karen participants highlighted a need for specific types of healthcare providers or health services, namely female obstetricians regionally, language-concordant aged care services and GPs in both settings. Participants also described long waits for appointments with healthcare providers or not being able to get an appointment, reflecting a lack of available services: “*we wait quite a long time to get an appointment… sometimes we cannot get an appointment*” (Chin, metropolitan). They also reported that when GP appointments were delayed on the same day, they were not always able to wait due to competing family responsibilities.

##### Subcategory 5.6 Lack of resourcing for community advocacy.

Metropolitan Karen participants, all of whom volunteered formally for their community in language and social support, service navigation, and advocacy capacities, highlighted the importance of volunteer-driven community initiatives to bridge gaps in support and interpretation services, translate health information, and advocate for women: “*we bridge for service gap*” (Karen, metropolitan). This was particularly important for women who were no longer eligible for settlement services, having lived in Australia beyond five years. They emphasized the financial burden advocacy placed on volunteers and advocated for more funding for volunteer organizations and paid support roles such as interpreting and case management, “*grants/$ to fully fund current services which are being paid by volunteers*” (Karen, metropolitan). They also recommended proactive community engagement in co-design of policies that affected their communities.

## Discussion

This study reports health information priorities identified by women of refugee background from Myanmar settled in WA and their community leaders. Initial categories, *Changing contexts* and *Motivations*, contextualize women's priorities, while *Information priorities*, *Information sources,* and *Structural barriers for action* identify health information needs and preferred sources of health information, with evident implications for service and policy initiatives. This discussion will explore the policy implications of women's recommendations.

The category, *Motivations,* affirms that women of refugee background from Myanmar settled in WA consider health as a priority in their lives. This aligns with prior research amongst refugee background people from Myanmar elsewhere in Australia (Wong et al., 2020). Identified information priorities largely reflect known health promotion needs, such as cancer, nutrition and SRH, amongst refugee background people from Myanmar and other countries during settlement (Davidson et al., 2024; Laverack, 2018; Wong et al., 2024). Participants demonstrated some knowledge about these topics; however, they were conscious of knowledge gaps and articulated the impact of these on their health behaviours. For example, women knew that regular screening was important for specific reproductive cancers; however, were unsure of the existence of other cancers. This suggests a lack of foundational knowledge needed to inform broader cancer prevention behaviours. Without that knowledge, women's ability to act proactively to promote their health is inhibited. The authors would suggest that more comprehensive health promotion campaigns and strategies are indicated to address women's identified priorities.

*Information sources* and *Structural barriers for action* situate women's information needs within a context of limited information resources and support. These findings affirm previous research on how women and people of refugee background from Myanmar access health information (Davidson et al., 2024; Griffin et al., 2022a; Wong et al., 2024). Participants' evaluation of information sources highlights gaps in availability and accessibility. Community networks were leveraged as sources of information sharing and support, while healthcare providers were both a trustworthy information source and support to appraise that information. However, these resources were constrained by structural factors including limited time and language-discordant communication. These too are previously reported barriers to health service access and navigation (Griffin et al., 2022b). The emphasis placed upon healthcare providers as both sources of information and support for appraisal of that information highlights the interdependent relationship between health literacy and health service access amongst women of refugee background from Myanmar. It shows that structural barriers influencing health service access and navigation similarly impede women's abilities to exercise their health literacy skills to build knowledge towards good health. Overall, these findings position health literacy as collective, relational and structurally constrained for women of refugee background from Myanmar in WA.

From their rapid review of health promotion for improved refugee and migrant health in Europe, Laverack (2018) recommends direct investment in structural interventions to improve refugee and migrant health through national policy. In WA, current state and national policies identify culturally and linguistically diverse populations as priority groups, outlining equitable approaches to health promotion and health literacy (Commonwealth of Australia & Department of Health, 2026; Department of Health & State of Western Australia, 2024; Western Australian Department of Health, 2019). The findings of this study highlight gaps in current health knowledge amongst women of refugee background from Myanmar, some directly aligned with identified priority areas in these policies, such as mental health and wellbeing (Commonwealth of Australia & Department of Health, 2026; Department of Health & State of Western Australia, 2024; Western Australian Department of Health, 2019). Gaps, particularly in regional communities, are also evident in the limited available health information resources, inadequate funding and resourcing of a key salutogenic resource, community networks, and inadequate access to and time with primary healthcare providers. If health literacy is understood to be a collective and relational resource amongst refugee background communities, as suggested from the findings of this and prior studies (Chao & Kang, 2020; Peprah et al., 2025), a strengths-based approach that builds on existing community networks may be warranted. Health promotion policy, initiatives, and information resources responsive to the ways that women of refugee background from Myanmar access, share, and use health information within communities are recommended.

The findings of this study indicate that greater investment in addressing these structural factors is required to improve access to and engagement with health information amongst women of refugee background from Myanmar in WA. While this study is specific to women of refugee background from Myanmar, prior studies have similarly identified structural barriers impeding health literacy, health promotion behaviours and health service access amongst other refugee background populations in Australia and globally (Hawkins et al., 2021; Mohr et al., 2023; Peprah et al., 2023; Taylor & Lamaro Haintz, 2017). From their qualitative exploration of how Australian primary health services respond to the health literacy and cultural needs of African people of refugee backgrounds, Peprah et al. (2023) concluded that greater policy attention may be required to address systemic barriers, as opposed to ad hoc service-driven interventions. Monani et al. (2021) proposed a nationally funded health communication service in each state and territory, tasked to map and evaluate health literacy strategies amongst migrant and refugee communities. The current global context of increasing conflict, forced displacement and negative public discourse about migration reinforces the need for a proactive approach to health responsive to the cultural, trauma, and politically informed needs of refugee background communities (Cherepanov, 2023). As identified by participants in the category *Changing contexts*, such troubling events can impact the mental health and wellbeing of refugee-background communities, impeding engagement with health-promoting activities and health services (Cherepanov, 2023; da Silva Rebelo et al., 2018). The findings of this study add to pre-existing evidence supporting consideration of more coordinated policy approaches prioritizing health literacy and health promotion amongst refugee-background communities in Australia. A statewide, if not national, health promotion strategy is perhaps indicated for refugee-background communities, responsive to relational and culturally mediated and structurally constrained health literacy practices along with the gendered nature of settlement and health experiences.

## Strengths and limitations

The PAR underpinnings are a study strength, including the design and findings informed by stakeholder reference group expertise, intended to promote collaboration and cultural safety (Vallianatos et al., 2015). On the other hand, the non-probability sampling method employed limits the representativeness of the sample and generalizability of findings; however, these are effective qualitative approaches to engage traditionally hard-to-reach populations (Bekteshi et al., 2024).

The selection of community listening sessions as a data collection tool is also a strength; the flexible nature of the sessions allowed for responsiveness to varying education and literacy levels (Ardoin et al., 2022) whilst drawing on the strength of collectivism characteristic of the Myanmar diaspora. However, it is possible that participants with diverging perspectives may have been reluctant to express these opinions in these sessions. The inclusion of a community co-convener and the composition of each session, bringing together women with shared cultural identity and language, were designed to promote trust and knowledge exchange. Only two sessions were audio-recorded, limiting the depth and comparability of the data collected in the other two sessions. Comprehensive field notes were taken to mitigate this, and presentation of preliminary findings to SRG members affirmed these interpretations. However, this may have resulted in differences in the depth of detail captured across sessions, as interactions between participants and between participants and the facilitator were captured to a limited degree in field notes. Similarly, participants' explanations of their responses and for their rankings were captured in more detail in the audio recordings. Multilingual data were collected. Professional interpreters and translators were engaged to mitigate the risk of translation errors.

During data analysis, women of refugee background from Myanmar and female community leaders were not treated as separate groups. Data were not collected in a way that consistently enabled the two to be distinguished. It is acknowledged that community leaders may have had different knowledge and perspectives than those not in formal leadership positions; however, community listening sessions were selected to promote dialogue and knowledge exchange between participants during the data collection process, well aligned with the collective nature of Myanmar diaspora communities.

While women of refugee background from Myanmar in WA were the focus of this study, detailed information about the study context and participant characteristics have been provided to support readers to assess the transferability of the study findings to other populations or settings.

## Conclusion

This study sought to report health information priorities and policy recommendations identified by women of refugee background from Myanmar in WA and their communities. Health information priorities, avenues for information delivery, and structural barriers are clearly identified. Women's recommendations are situated within the changing contexts of their lives and their motivations for good health, key aspects that women felt service providers and policymakers needed to understand. Overall, findings suggest that, for this population, health literacy is collective, relational and constrained by identified structural barriers. A coordinated health promotion infrastructure, underpinned by stronger policy and investment, is suggested to support improved information and health service access, interpretation support, funded community advocacy, and to minimize transport and affordability barriers, to meet the needs of women of refugee background from Myanmar in WA.

## Supplementary Material

Supplementary Material Supplementary Material.docx

## Data Availability

The data are not publicly available due to ethical and privacy reasons.
