# Supplementary material for: Health information priorities and recommendations: findings from community listening sessions with women of refugee background from Myanmar in Western Australia
Source: Int J Qual Stud Health Well-being. 2026 Sep 9;21(1):2729863. doi: 10.1080/17482631.2026.2729863 (PMC13560413; doi:10.1080/17482631.2026.2729863)
Supplement: Supplementary file 1 [file ZQHW_A_2729863_SM1042.docx]

**Supplementary Material**

**To the article:** Health information priorities and recommendations: Findings from community listening sessions with women of refugee background from Myanmar in Western Australia

**Brief Demographic Questionnaire**

1. How old are you? ______
2. What year did you arrive in Australia? ­___________
3. What is the primary language you speak at home? ________________
4. How well can you read in your primary language?
   - Not at all
   - Not well
   - Well
   - Very well
5. How well can you write in your primary language?
   - Not at all
   - Not well
   - Well
   - Very well
6. How well can you read in English?
   - Not at all
   - Not well
   - Well
   - Very well
7. How well can you write in English?
   - Not at all
   - Not well
   - Well
   - Very well
8. Do you require an interpreter at health appointments?
   - Yes, always
   - Yes, sometimes
   - No
9. Are you the parent or carer of a child/children in Australia?
   - Yes
   - No

10. Please describe your employment status:

	Paid work, full-time

	Paid work, part-time

	Paid work, casual

	Paid study or scholarship

	Unpaid work (e.g. volunteering)

	Homemaker

	Other: ____________________

11. What is your highest level of education?
    - No schooling
    - Primary school
    - High school
    - TAFE
    - University degree
    - Other: ____________________

**Community Listening Sessions – Semi-Structured Question Guide**

*Please note that this guide is to be used in conjunction with the Community Listening Sessions Facilitation Plan. Prompts for each question are presented in italics below, to be asked if needed to elicit discussion.*

**Being Healthy and Health Information**

In our research, we have explored health information. Health information is information about staying healthy, treating illness, finding out about a symptom, or finding out about a health service or healthcare provider like a doctor or nurse.

**Q1 What role does health information play in being healthy?**

- *Where/who does health information come from?*
- *How have you used information to help you stay healthy or when you have been unwell or to help a woman of refugee background from Myanmar to stay healthy or when they have been unwell?*
- *Can you tell us more about that?*
- *Can you describe what you have drawn?*

We interviewed women about their experiences looking for and making sense of health information in Australia. From their experiences, we made this model to represent their health information experiences.

*Present laminated model in English, Burmese, Karen or Chin as appropriate.*

This is a set of skills called health literacy. Health literacy can be broken down into these steps:

(1) identifying that you need health information;

(2) looking for it;

(3) accessing it;

(4) understanding the information;

(5) applying it to your own situation and health and life;

(6) using it in a meaningful way that help you be healthy.

The women we interviewed described barriers to each of these steps. They also described how their experiences before coming to Australia influenced how they expected to be able to find health information in Australia. They also described asking someone in the community, maybe a community leader or a friend who has lived here longer, to help them with their health. When the women had more experience with healthcare and health problems in Australia, they started to help other women. Other times their own health needs could become more important than helping other people. That is what the model represents.

**Q2 How does this model match your experiences?**

- *Is there anything you would change?*
- *Can you show us on the model?*
- *Can you tell us more about that?*

**Health Information Priorities**

**Q3 What health topics are important for women of refugee background from Myanmar to have information about?**

- *List these important topics*
- *Can you explain why these topics are important?*
- *What about these health topics do women need information on or to know more about?*

**Q4 Are some topics more important than others?**

- *Can you explain why?*
- *Can you tell us more about that?*
- *How would you rank these priorities from most to least important?*

**Q5 What is the best way to get information to women?**

- *What would make it easiest for women to find and access health information?*
- *What would make it easiest for women to understand health information?*
- *What would make it easiest for women to use health information?*
- *Can you tell us more about that?*

**Service and Policy Recommendations**

**Q6 What skills would help women of refugee background from Myanmar to have good health and wellbeing?**

- *What skills would help them/you to be healthy?*
- *What skills would help them/you look for, access, understand or use health information?*
- *Can you speak more about that?*

**Q7 What resources would you like to help you to have good health and wellbeing?**

- *What resources or support from services or government would help you to stay healthy?*
- *What resources would help you look for, access, understand or use health information?*
- *Can you speak more about that?*

**Q8 What would you like service providers to know about how to help you and women in your community find, access, understand and use health information?**

- *What do you think governments could do to make this easier?*
- *What do you think service providers could do to make this easier?*
- *Can you describe that?*
- *Can you explain what you mean?*

**Q9 What would you like policymakers to know about how to help you and women in your community find, access, understand and use health information?**

- *What do you think governments could do to make this easier?*
- *What do you think service providers could do to make this easier?*
- *Can you describe or explain that?*
